# The Resistance of Polyethersulfone Membranes on the Alkaline Cleaning Solutions

**DOI:** 10.3390/membranes14020027

**Published:** 2024-01-23

**Authors:** Marek Gryta, Piotr Woźniak

**Affiliations:** Faculty of Chemical Technology and Engineering, West Pomeranian University of Technology in Szczecin, ul. Pułaskiego 10, 70-322 Szczecin, Poland; piotr.wozniak@zut.edu.pl

**Keywords:** PES, PVP, ultrafiltration, car wash, alkaline solution

## Abstract

Polyethersulfone (PES) is a polymer popularly used to produce ultrafiltration (UF) membranes. PES is relatively hydrophobic; thus, hydrophilic ingredients are added to the membrane matrix to reduce the fouling intensity. Ingredients such as polyvinylpyrrolidone (PVP) reduce the resistance of PES to NaOH solutions. This study investigated the possibility of using PES membranes for the separation of alkaline cleaning solutions. For this purpose, self-made PES membranes and commercial ultrafiltration PES membranes (UE10—10 kDa and UE50—100 kDa) containing PVP additive were used. The membranes were soaked for 18 months in alkaline (pH = 11.3–11.5) solutions of car washing fluids. It has been found that long-term contact with these solutions caused changes in the structure of the surface layer, especially of membranes containing PVP. As a result, the separation of dextran (100–200 kDa) decreased by 30–40% for PES membranes, 30–40% for UE10 and 40–60% for UE50. Despite these changes, the separation efficiency (rejection of COD, NTU and anionic surfactants) of synthetic car wash wastewater (mixture of surfactants and hydrowax) was similar to the results obtained for pristine membranes.

## 1. Introduction

Polymeric membranes are often used for the water and wastewater treatment and separation of solutions in food and biotechnological processes [1,2,3,4]. Polyethersulfone (PES) is one of the most promising membrane materials used in the ultrafiltration (UF) of water [5,6,7,8,9]. PES has both excellent chemical and thermal resistances over a wide range of pH, from 2 to 12 [1,10,11]. For this reason, PES membranes have also been successfully used to treat wastewater from a car wash, where a cleaning agent with a different pH value was used [11]. However, PES is relatively hydrophobic, which may accelerate the membrane-fouling phenomenon [9,12]. To reduce fouling during the production of membranes, ingredients that increase the hydrophilicity of PES membranes, such as polyvinylpyrrolidone (PVP) [1,13,14], are added to casting solutions. Fouling is one of the main reasons that make the application of membrane processes difficult. Acid and alkali solutions are often used to remove deposits from membrane surfaces [6,8,15]. Effective cleaning using alkali (e.g., NaOH) of fouled PES membranes has been demonstrated in many studies [11,12,16]. The cleaning strategies recommended for PES membranes are usually as follows: contact times of 20–40 min for alkaline and 15–30 min for acidic cleaners and temperature in the range of 303–323 K. The contact time of cleaning solutions is relatively short; therefore, cleaning procedures usually do not cause significant damage to membranes [10,16]. However, repeated cleaning may cause changes in the PES structure [10]. High efficiency in membrane cleaning was also achieved by using sodium hypochlorite (NaClO) [10,17]. However, this strong oxidant is responsible for the formation of free radicals, resulting in chain scission of PES molecules. Therefore, such cleaning solutions may damage the membrane structure and shorten the membrane lifespan. Many studies have demonstrated the negative impact of NaClO solutions, especially if PES membranes contained PVP [1,8,14,18,19]. PVP was oxidized and dislodged due to NaClO aging, which also caused the chain scission of PES, especially at a solution with a pH over 9 [18,19]. PES hydroxylation was observed only for membranes containing PVP, the hydroxylation rate being dependent on the PVP content [1]. Hence, the presented results indicate that the addition of PVP, which improves the performance of PES membranes, may also limit the scope of their application. It has been shown that damage in PES membranes can be repaired using a colloidal gel solution, which was entrapped in the damaged site after filtration deposition [20]. Microgel-healed PES UF membranes are additionally resistant to cyclic washing with NaOH solutions.

A number of industrial cleaning agents used to clean membrane installations contain NaOH [1,10,21,22]. Performing the literature review allowed us to confirm that NaOH was efficient in cleaning the PES membrane, which is also used in water treatment. Cleaning with such solutions removes protein and oil impurities, and only a small number of adsorbed impurities remain on the membrane surface [11,12]. Typically, periodic cleaning of membranes takes a relatively short time, limiting the negative impact of the chemical cleaning agents used [10]. If the UF process is used to treat alkaline wastewater, membranes that are also resistant to long-term contact with alkaline solutions should be used. A high resistance of UF membranes (150 kDa) to NaOH solutions was demonstrated in [10]; however, the presented results were obtained from short experimental tests. In turn, in the work by [6], the impact of NaOH and HNO_3_ solutions on six types of PES membranes (4–20 kDa) was tested for 150 days, and no serious damage was found. Nevertheless, significant changes in the surface structure of the membranes washed using alkaline solutions, especially those containing PVP, were found in other studies [23,24]. It has been documented that the cleaning agents can remove PVP from membranes, leading to a change in the structure of the membrane surface layer [19,22]. Due to the presented discrepancies in the durability of PES membranes, the development of industrial applications requires further, long-term research. This conclusion was also presented in [25], in which 15 membrane washing cycles were performed, and changes in membrane performance were observed after more than five cycles.

Various cleaning solutions are used to wash cars, including alkaline cleaning agents [26]. For this reason, chemically resistant membranes should be used to treat wastewater from car washes. It has been shown that a PES membrane can be used to separate such wastewater, and the obtained high-quality permeate could be used as re-use water for washing cars [11]. To implement this idea, however, it is necessary to know whether the separation of wastewater from a car wash will not damage the membranes. For this reason, long-term tests of the PES membrane resistance to alkaline car-washing agents were carried out.

## 2. Materials and Methods

### 2.1. Membranes

In the present study, the self-made PES membranes and two types (UE10 and UE50) of commercial ultrafiltration PES membranes were used. The commercial membranes were manufactured by TriSep Corporation (Goleta, CA, USA). The nominal molecular weight cut-offs (MWCOs) declared by the manufacturer were 10 kDa and 100 kDa for the UE10 and UE50 membranes, respectively.

The commercially available ultrafiltration PES membranes may contain PVP [1,14,19]. For this reason, for comparison, membranes made of pure PES were also used for testing. The self-made PES membranes were synthesized by the wet phase inversion method, using PES as polymer, N,N-dimethylformamide (DMF) as a solvent and ultrapure water as a non-solvent. Granulat PES (Ultrason E6020P) was provided by BASF SE (Ludwigshafen, Germany), and DMF was acquired from Avantor Performance Materials Poland SA. The casting solution (15% PES) was cast as a thin film on a glass plate using an automatic applicator (Elcometer 4340, Elcometer Ltd., Manchester, UK), with the casting knife gap set at 0.1 mm. The cast film was then immersed in an ultrapure water coagulation bath at 293 K for 24 h.

### 2.2. Tests of Membrane Aging

The long-term resistance of the tested PES membranes to alkaline solutions (pH = 11.3–11.5) was determined using the static method. Membrane samples (7 × 7 cm) were placed in containers (0.2 L) filled with solutions (0.3% and 0.5%) of cleaning agents commonly used for cars washing cars. These agents are produced in the form of concentrated solutions that are dosed into the process water at the car wash. In the conducted research, the agent concentrates were diluted to the level corresponding to those used in a car wash. The composition of these concentrates is presented in Table 1. The containers were stored in the dark at room temperature for 18 months. Five membrane samples were placed in the containers, and each type of container was duplicated. Periodically, the membrane sample was removed, and after rinsing in ultrapure water, its separation properties and surface structure were examined.

In addition, the resistance of the membranes to pure NaOH solutions (3 g/L and 1 M) was tested. Tests on the resistance of PES membranes to NaOH solutions have also been carried out in other studies [6,24]. In order to compare the obtained results, solution concentrations were used in accordance with those used in the above-mentioned works. In this case, the membranes were immersed in the NaOH solution for 1 h, 1 d and 7 days. Then, the membranes were rinsed with ultrapure water. 

### 2.3. UF Studies

**Figure 1:**
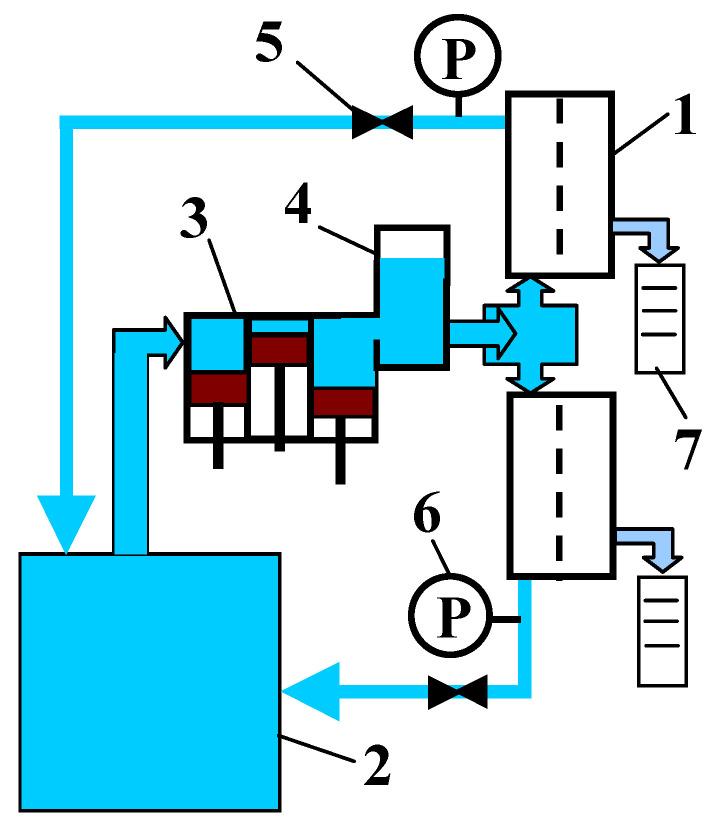
UF installation with two plate modules. 1—module, 2—feed tank, 3—pump, 4—pressure dampener, 5—needle valve, 6—manometer, 7—measurement cylinder.

The separation properties of UF membranes are usually characterized by the rejection of molecular markers, such as polyethylene glycols or dextran [6,17,24]. In this work, changes in separation performance caused by an alkaline cleaning solution were determined on the basis of changes in the degree of dextran rejection. The UF installation shown in Figure 1 was used for these tests. This was a laboratory-scale membrane installation consisting of a piston pump with a pressure dampener, two stainless-steel membrane modules, a manometer and a needle valve connected in parallel. The working area of each membrane was 0.0025 m^2^. 

The UF experiments were carried out at the transmembrane pressure (TMP) of 0.1 MPa. The applied feed flow velocity was equal to 1 m/s. The feed, after flowing through the module, was returned to the tank. Changes in membrane permeability were measured for distilled water at the TMP in the range of 0.1 to 0.3 MPa. During the investigations, the feed temperature was between 293 and 295 K.

### 2.4. Analytical Methods

The studies on dextran separation were performed with the use of their solutions at a concentration of 0.5 g/L. For this purpose, dextran with a molar mass within the range of 70 and 500 kDa produced by Polfa (Łódź, Poland) was used. A dextran concentration both in the feed and permeate was determined using a high-performance liquid chromatograph (HPLC), model UlitiMate 3000 (Dionex, Sunnyvale, CA, USA), with refractometer RI-101 (Shodex) and PolySep-GFC-P 4000 column (Phenomenex, Torrance, CA, USA), through which DI water flowed (0.8 mL/min). The dextran rejection efficiency R [%] was determined as follows:

(1) $$R=\frac{C_{F}{\;-\;C}_{P}}{C_{F}}100\%$$

where C_P_ [mg/L] and C_F_ [mg/L] are the measured dextran concentrations of the permeate and feed, respectively. Initial feed volume was 3 L. Permeate samples were collected after a 15 and 35 min duration of the UF process.

In addition to dextran, the rejection of components of synthetic wastewater containing 0.5% detergents (Euro Turbo Foam) and 0.2% hydrowax was investigated. These agents are used for washing cars, and their composition is given in a previous work [11]. The turbidity of this mixture was 4.8 NTU, the chemical oxygen demand (COD) was 3690 mg O_2_/L and the concentration of anionic surfactants was 702 mg/L. To determine the values of these parameters, the Hach cuvette tests (DR2800 spectrophotometer, Hach Lange, Düsseldorf, Germany) were used (LCK 1014—COD, and LCK 344—anionic surfactants). The turbidity was determined using a portable turbidimeter, model 2100 AN IS (Hach Company, Loveland, CO, USA).

In order to identify the functional groups on the membrane surfaces, the attenuated total reflection Fourier transform infrared (ATR-FTIR) analyses were performed using a Nicolet 380 FTIR spectrophotometer, coupled with Smart Orbit diamond ATR accessory (Thermo Electron Corp., Austin, TX, USA). Spectra from 400 to 4000 cm^−1^ were collected by accumulating 32 scans at a resolution of 4 cm^−1^. The PVP (40 kDa) purchased from Sigma Aldrich Chemicals (Poznań, Poland) was also used for FTIR studies.

The studies of membrane morphology and surface composition were performed using a Hitachi SU8000 Field Emission Scanning Electron Microscope (FESEM) with an energy-dispersive X-ray spectrometer (EDS) (Hitachi, Tokyo, Japan). All the samples were sputter-coated with chromium. Samples for cross-sectional testing were fractured in liquid nitrogen. The membrane surface was also examined using atomic force microscopy (AFM). A multi-mode 8 AFM apparatus equipped with a Nanoscope V converter from Bruker (Santa Barbara, CA, USA) characterized the membrane roughness in the scanasyst mode. The Ra and Rq parameters were evaluated on the basis of at least five AFM images (10 μm × 10 μm).

Soaked membrane samples, after rinsing in ultrapure water, were dried by three-step rinsing in ethanol solution (24, 48 and 96%) to remove residual water before the ATR-FTIR, AFM and SEM studies. Wet membrane samples or samples dried in hot air (363 K) were also used for the ATR-FTIR measurements.

## 3. Results and Discussion

### 3.1. Membrane Performance

**Figure 2:**
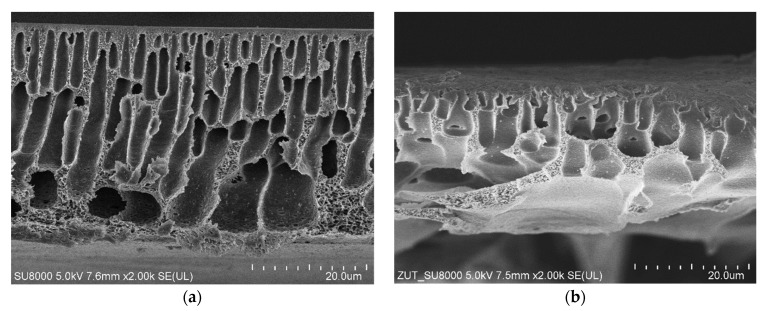
SEM images of UF membranes cross-section: (**a**) self-made PES membrane, (**b**) UE50 commercial PES membrane.

The UF process typically uses an asymmetric membrane that features a thin and dense separation layer supported by a finger-like pore membrane bulk. The performed SEM observations confirmed that the PES membranes used in the present work also had such a structure (Figure 2). The image of the UE10 membrane was similar to that shown in Figure 2b. The thickness of the tested membranes was 30–40 μm. The outer separation layer in the membranes was very thin (below 1 μm), which may favor its damage.

**Figure 3:**
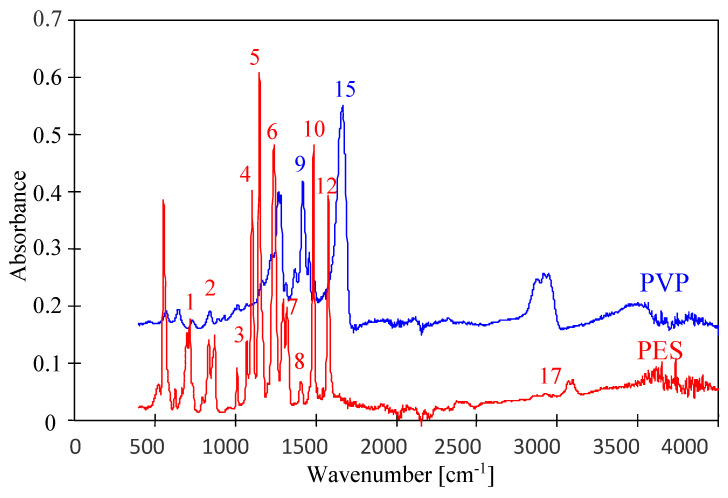
ATR-FTIR spectra of pristine self-made PES membrane and PVP powder.

**Figure 4:**
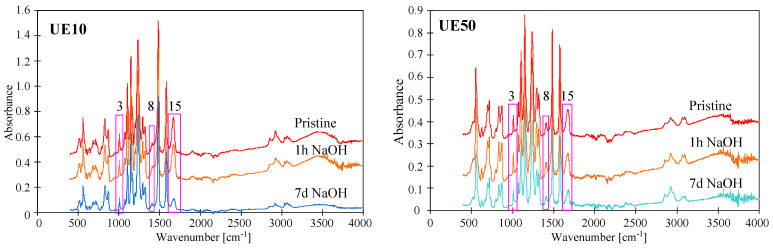
ATR-FTIR spectra of pristine commercial PES membranes (UE10 and UE50) and these membranes aged with NaOH solution (3 g/L) at different soaking times (1 h and 7 days).

The tested membranes contained the same main component; hence, similar ATR-FTIR spectra were obtained (Figure 3 and Figure 4) with peaks characteristic of PES. In the figures, they are marked with numbers corresponding to Peak No from Table 2. To obtain the distribution of PES-specific peaks, the respective IR band was integrated into the wave–number range of 1560 cm^−1^ to 1597 cm^−1^, whose assignment corresponds to C=C ring vibration, 1298 and 1239 cm^−1^ (C–O–C stretching vibrations). For the PVP distribution, it was integrated in the range of 1635 cm^−1^ to 1695 cm^−1^, whose assignment corresponds to C=O bound [1]. This last peak was not present on the spectra of self-made PES membranes (Figure 3); however, ATR-FTIR spectra obtained for UE10 and UE50 membranes had it (Figure 4, No. 15). This finding proves that the tested commercial PES membranes contain PVP additives. It was found that this absorption band is attributed to the C=O amide bound of PVP progressively disappearing during the PES/PVP membrane aging [14].

It is important to note that commercial PES membranes generally show good resistance to NaOH solutions [6]. However, some works indicate that the addition of PVP reduces the resistance of PES membranes to alkaline solutions [14,19]. This is due to the degradation of PVP, whose loss from a pristine membrane was observed on FTIR spectra as a decrease in the intensity of a band located at 1661 cm^−1^ [14], marked in this work as No. 15 (Table 2). The tests carried out showed that the degradation effects are a function of the soaking time, which was also observed in another study [22]. Membranes soaked for 1 h in a NaOH solution had FTIR spectra similar to those obtained for pristine membranes (Figure 4). Similarly, no visible signs of degradation of the top layer by dilute NaOH solutions were found in [10]. However, after 7 days of soaking the membranes, there was a significant reduction in the intensity of peak No. 15. In the work by [22], the complete disappearance of PVP was found after membrane washing using NaOH for 2 weeks. Importantly, these results confirm that PES membranes are resistant to short-term washing using diluted NaOH solutions; however, they may be damaged during the long-term separation of alkaline solutions.

**Figure 5:**
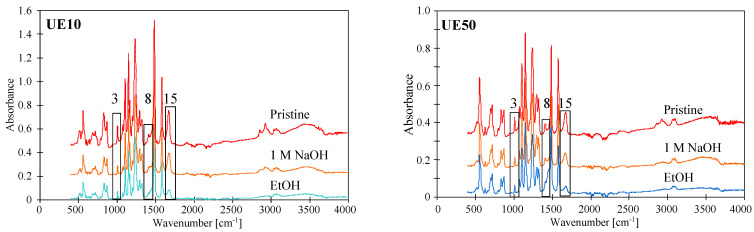
ATR-FTIR spectra of commercial PES membranes (UE10 and UE50) soaked for 24 h in a 1 M NaOH solution followed by ethanol rinsing (EtOH).

The rate of PVP degradation is also influenced by the concentration of the alkaline solution. Increasing the NaOH concentration from 3 g/L to 1 M (pH = 14) decreased the intensity of the No. 15 peak after 24 h of contact (Figure 5). It should be pointed out that a similarly strong effect of 1 M NaOH was also found in several other studies [1,14,24,28]. Teella et al. [16] connected this to the base-catalyzed hydrolysis of the lactam ring in additives of PVP. After soaking in the NaOH solution, the intensity of the No. 15 peak further decreased after rinsing the membrane with ethanol (EtOH). This should be attributed to the NaOH-catalyzed hydrolysis, which led to the breakage of some polymer chains, so the relatively loose surface structures were removed using EtOH [28]. The leaching of components from the surface was also confirmed using SEM-EDS analysis (Table 3). Compared with the pristine membranes (UE10 and UE50), the surface composition of both membranes changed after rinsing using NaOH and EtOH solutions.

FTIR-ATR spectra presented in Figure 4 and Figure 5 allow us to demonstrate the influence of contact time and NaOH concentration on the degradation of PES. Peak located at 1030–50 cm^−1^ (No. 3) is commonly attributed to an evolution of the PES skeleton itself; thus, this peak is a signal revealing the PES degradation [14]. The intensity of this peak progressively decreased when contact time was increased to 7 days (Figure 4). An increase in the NaOH concentration to 1 M caused a similar effect to occur after 24 h of soaking the membranes (Figure 5). On the presented FTIR-ATR spectrum, one can also notice the disappearance of the peak at 1320 cm^−1^ (No. 8), which is attributed to Ar–SO_2_–Ar asymmetric stretching vibrations [6,14]. Decreasing the intensity of this peak indicates the breakage of some polymeric chains.

### 3.2. Membranes Soaked in Alkaline Solutions

In addition to surfactants, cleaning agents used for car washing may contain NaOH (Table 1). For this reason, wastewater generated in the car wash will be alkaline, which may damage the PES membranes used for their separation. In order to investigate their resistance, the membranes were soaked for several months in Wheel and Insect solutions at the concentration used for washing cars (0.3–0.5%) and with a pH value of 11.3–11.5.

**Figure 6:**
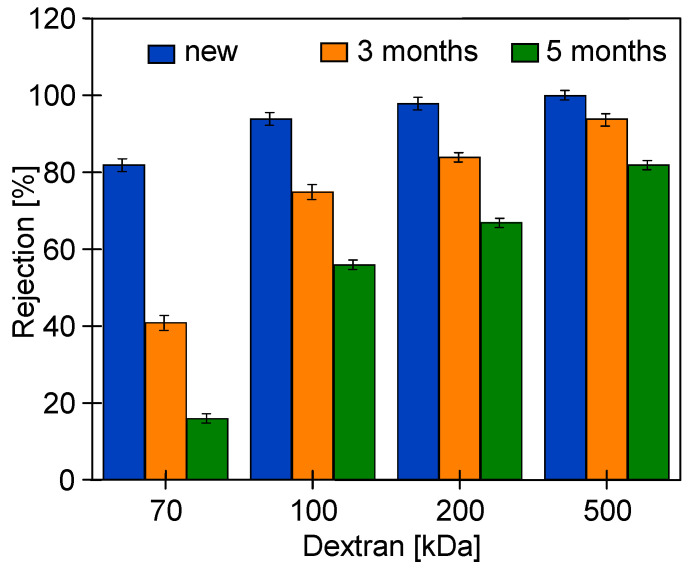
Dextran rejection by the studied self-made PES membranes. Membranes soaked into an Insect 0.5% solution up to a 5 month period.

**Figure 7:**
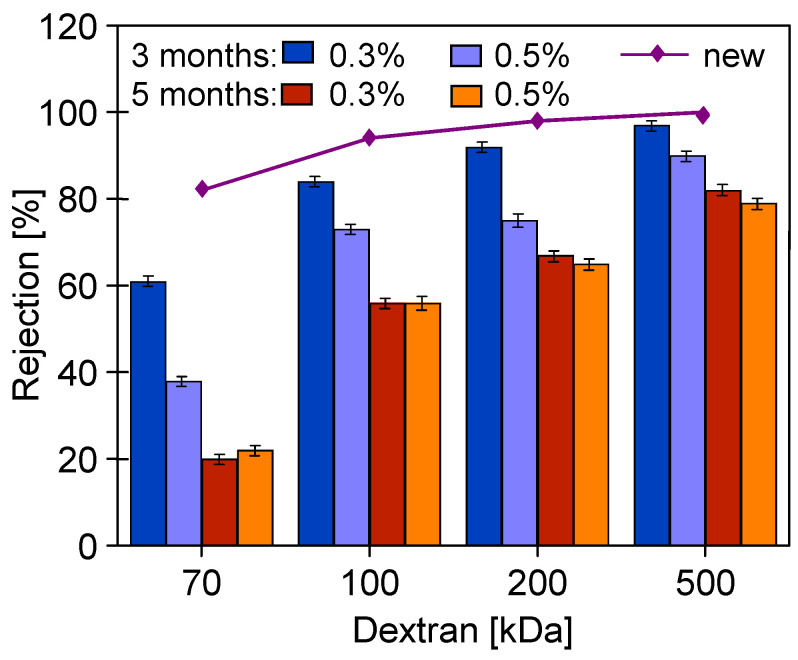
Dextran rejection by the studied self-made PES membranes. Membranes soaked into 0.3% and 0.5% Wheel solutions for up to a 5 month period.

Usually, the degradation of UF membranes leads to an increase in the permeate flux and a decrease in the separation degree [19]. Hence, in this work, dextran with different molecular weight (MW) values were used to study separation changes. The results obtained for self-made PES membranes are shown in Figure 6 and Figure 7. Pristine membranes retained 100 kDa dextran by 94%. After 3 months of contact with the Insect solution, this value decreased to 75%, and after 5 months of soaking, it decreased to 56%. Similarly, significant decreases in the degree of retention were recorded for dextran with higher MW values. In the case of 500 kDa dextran, after 3 months of soaking, the rejection decreased from 100 to 94%, and after 5 months, it decreased to 82%.

Membranes soaked in the Wheel cleaning solution showed separation reductions in magnitude similar to those reported for Insect (Figure 7). In this case, dextran 100 kDa was rejected by 73% and 56% after 3 and 5 months, respectively. Decreasing the concentration of Wheel fluid from 0.5 to 0.3% reduced the negative impact, however, only during the first 3 months of soaking. The results presented in [6] demonstrate that the greatest changes in the structure of PES occurred in the initial period of contact with NaOH solutions. Other studies lasting 180 days showed that after 60 days of contact with alkaline solutions, the polymer properties stabilized [29]. These results indicate that there are probably fewer resistant places in PES membranes that are degraded in the initial period of NaOH exposure. Increasing the concentration of the solution accelerates this effect (Figure 5), and after 3 months of soaking, dextran rejection was lower for the 0.5% solution (Figure 7). In the next period, this difference disappeared because the remaining places were more resistant to NaOH. For this reason, after soaking the membranes in 0.3% and 0.5% solution for 5 months, the differences in dextran rejection obtained were insignificant and depended mainly on the contact time. Remarkably, this result indicates that even dilute solutions may cause negative changes in the structure of PES membranes during their long-term industrial use.

**Figure 8:**
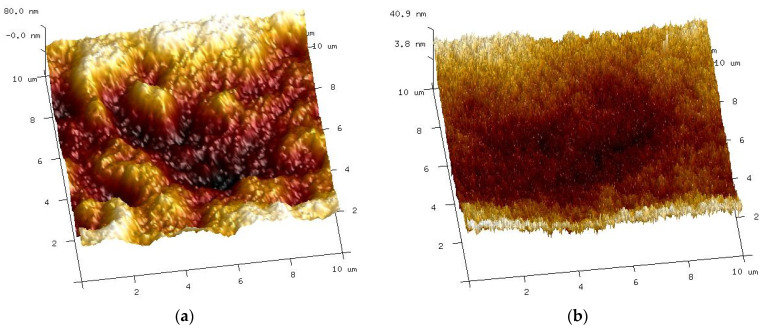
AFM image of surface self-made PES membranes: (**a**) pristine and (**b**) soaked for 5 months in a 0.5% Insect solution.

The removal of preservatives (e.g., glycerol and PVP) from the chemically pretreated membranes, which causes pore enlargement, is usually indicated as the reason for the deterioration of dextran rejection by commercial UF membranes [24]. In the case of the tested self-made PES membranes, there was no addition of PVP or other preservatives. This suggests that there were other reasons for the pore enlargement. Hence, it can be clearly indicated that this phenomenon was due to the PES chain scission caused by the action of NaOH [1,9,18]. AFM tests showed that the surface of the soaked membranes became smoother (Figure 8). As a result, its roughness was reduced, and the value of the Rq parameter decreased from 24 to 7.2 nm. According to the aforementioned data, it can be concluded that the used alkaline solutions caused changes in the surface morphology.

**Figure 9:**
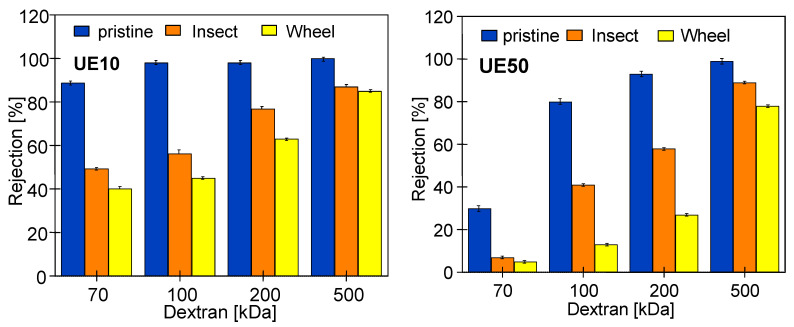
Dextran rejection by commercial PES membranes (UE10 and UE50). Membranes soaked for 5 months in 0.5% Insect and Wheel solutions.

The commercial membranes used for the study had a claimed MWCO of 10 kDa (UE10) and 100 kDa (UE50). Therefore, dextran rejection by pristine UE10 membranes was much higher (Figure 9). However, in the conducted studies, rejection close to 100% was obtained for values higher than the declared ones, i.e., 100 kDa for UE10 and 200 kDa for UE50. The MWCO values higher than those claimed by the manufacturer for PES membranes were also found in the work by [24]. In the study by [6], it was found that the difference was due to the concentration polarization, especially at higher fluxes, which facilitated the penetration of dextran into the permeate.

A reduction in dextran rejection as a result of soaking the membranes in alkaline solutions was also observed for the tested commercial PES membranes. The presence of PVP in UE10 and UE50 membranes was confirmed using FTIR studies (Figure 5). Literature data indicate that adding PVP to the PES membrane increases their degradation under the influence of NaOH solutions [1,14,19]. However, the results presented in Figure 9 do not indicate that these membranes were degraded significantly more than self-made PES during 5 months of soaking in Insect and Wheel solutions. The UE10 membrane soaked in Insect rejected the 100 kDa dextran at 53% and the 200 kDa dextran at 75%. These values are similar to those obtained for a pure PES membrane (Figure 6). Slightly lower rejection values were obtained for UE50 due to the fact that this membrane had larger pores (MWCO 100 kDa). Notably, this membrane also showed much greater degradation in the Wheel solution.

**Figure 10:**
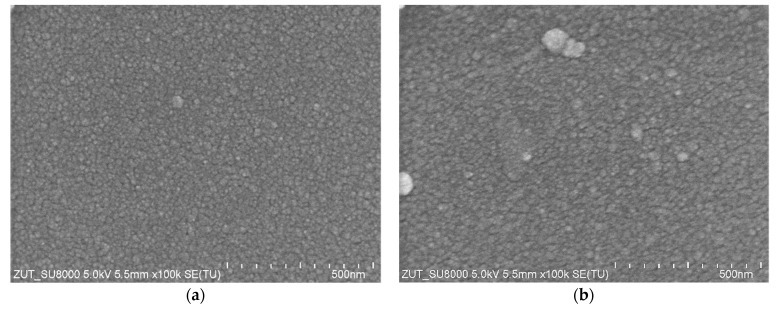

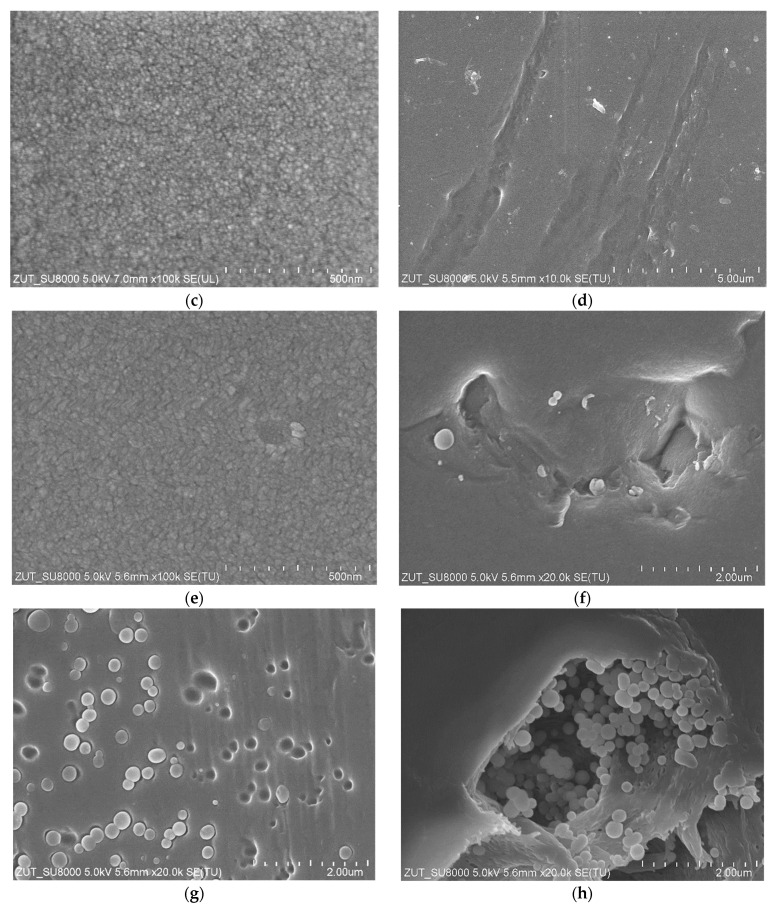
SEM images surface of commercial UE membranes soaked in a 0.5% Insect solution: (**a**) pristine UE10, (**b**) UE10 soaked for 5 months, (**c**) UE10 soaked for 10 months, (**d**) soaked UE10—linear depressions, (**e**) pristine UE50, (**f**) UE50 soaked for 5 months, (**g**) PVP spherical agglomerates on the UE50 surface and (**h**) large pore filled with PVP agglomerates.

The results of the SEM examination of the surface of membranes soaked for 5 months are shown in Figure 10. The pores in UF membranes are small; hence, they were not visible even at a magnification of 100k. The image of the surface of UE10 membranes soaked for 5 months (Figure 10b) was similar to that observed for pristine membranes (Figure 10a) and did not change after 10 months of soaking (Figure 10c). Only in a few places on the surface were changes in the form of longitudinal depressions noticed (Figure 10d). Significantly greater changes were observed on the UE50 membrane surface. Macro-changes occurred locally on its surface, as shown in Figure 10f. Particularly interesting was the appearance of spherical particles with a diameter of 0.2 μm and smaller in several places (Figure 10g). There were also several pores nearby. The similarity in the size of spherical particles and pores indicates that the pores were created by removing the particles. The formation of similar spherical particles was observed due to the agglomeration of PVP blended inside another polymer [30]. Most likely, during the production of PES membranes, some of the added PVP (hydrophilic) did not mix with PES (hydrophobic) and agglomerated into the observed spherical particles that were incorporated into the surface layer. A non-homogeneous accumulation of PVP inside the PES membrane top surface has also been demonstrated [1]. Due to the action of the alkaline solution, the thin PES layer covering the PVP agglomerates disappeared. The weakening of such a place is also evidenced by the SEM image presented in Figure 10h, showing that in the place where a large number of PVP agglomerates accumulated, the membrane surface layer disappeared over an area of several micrometers. The UE50 membranes had larger pores (100 kDa), which was probably achieved by the greater addition of PVP to the casting solutions [1]. As a result, the alkaline solution damaged the UE50 membranes to a greater extent. It should be pointed out that despite such significant damage, the degree of dextran retention did not decrease dramatically. The gel layer formed by the dextran probably penetrated these pores, closing them. A similar solution for repairing the damage to the PES membranes was presented in the study by [20], in which microgels were used as the healing materials.

**Figure 11:**
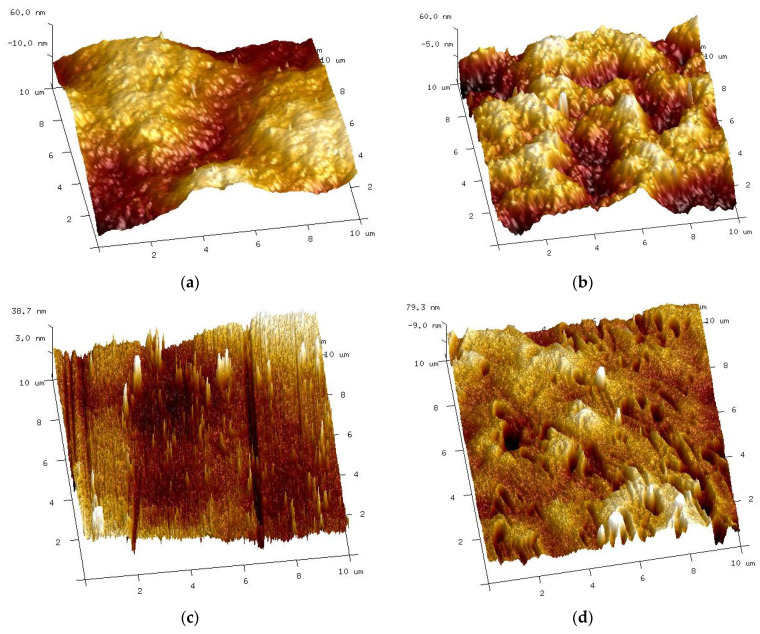
AFM images of the surface of commercial UE10 and UE50 membranes, pristine ((**a**)—UE10 and (**b**)—UE50), and membranes soaked for 5 months in a 0.5% Insect solution: (**c**) UE10 and (**d**) UE50.

Various changes in the surface of UE10 and UE50 membranes under the influence of alkaline solutions were also confirmed by AFM studies (Figure 11). The UE10 membrane became smoother after soaking it for 5 months. Conversely, numerous pores were revealed on the UE50 surface, corresponding to those observed during the SEM studies (Figure 10g).

### 3.3. Long-Term Studies

The results obtained after soaking the membranes in alkaline solutions for 5 months showed significant changes in the structure of their surface. However, industrial membranes are used for a much longer period of time. For this reason, the soaking of the membranes was extended to 18 months to determine whether the membrane’s degradation would increase in the following months.

**Figure 12:**
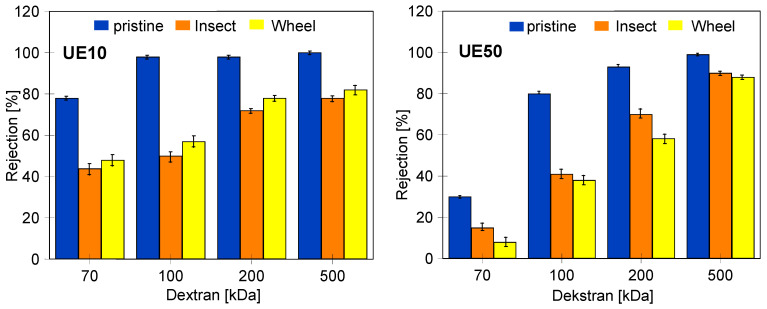
Dextran rejection by commercial UE10 and UE50 membranes. Membranes for soaked 18 months into 0.5% Insect and Wheel solutions.

**Figure 13:**
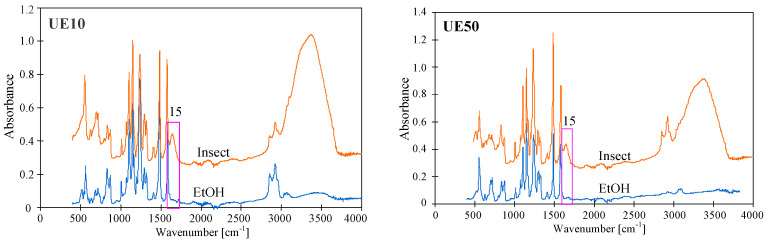
ATR-FTIR spectra of commercial PES membranes (UE10 and UE50) soaked for 18 months in a 0.5% Insect solution (pH = 11.5) and these samples after drying using ethanol solutions (EtOH).

The performed UF tests showed that despite a three-fold increase in the contact time of the membranes with alkaline solutions, there was no deterioration in dextran rejection (Figure 12). For example, the UE10 membrane soaked in Insect rejected the 100 kDa dextran at 50% and the 200 kDa dextran at 72%. These values are only 3% lower than those obtained after 5 months of soaking (Figure 9). This result indicates that alkaline solutions caused degradation of the tested PES membranes mainly in the initial period. During this period, PVP was leached from the membrane matrix due to its low resistance to NaOH solutions [1,9,14]. Significant PVP losses were detected after 7 days of membrane soaking (Figure 4, peak No. 15). In membrane samples removed from the alkaline solution after 18 months, FTIR analysis still showed the presence of the No. 15 peak characteristic of PVP (Figure 13); however, it was no longer present in membranes dried by rinsing them in an EtOH solution (without stirring). This finding indicates that, in places containing PVP, there is a breakage of some polymeric chains, and the relatively loose structures created were removed by EtOH [28]. SEM observations confirmed that there were no longer as many spherical PVP agglomerates on the membrane surface as was observed in the samples analyzed after 5 months of soaking (Figure 10g).

The results (Figure 12) obtained for samples soaked in the Wheel solution are interesting, as they show an increase in dextran rejection compared with the results obtained after 5 months (Figure 9). In the case of the UE10 membranes, the rejection (Wheel) was 5–7% higher than that obtained for samples soaked in the Insect solution. However, much greater increases were found for UE50 membranes; for example, for 100 kDa, the rejection increased from 13 to 38%, and for 200 kDa, it increased from 27 to 58% (compared with the data in Figure 9). The improvement in the separation of fouled PES membranes periodically washed with NaOH solution was also presented in [16]. This is influenced by changes in the membrane structure, which depend on both the composition of the solution and the contact time [8,22]. The manufacturer’s data (Table 1) show that the Wheel fluid additionally contained 1-Propanaminium, 3-amino-N-(carboxymethyl)-N,N-dimethyl-,N-(C12-18 (even numbered) acyl) derivs. It is an amphoteric surfactant that has a synergistic effect with anionic surfactants. As a result, it increases the foaming effect and is popularly used in cosmetic lotions for washing the body and hair. This difference in composition did not cause significant differences in the FTIR test results, and the obtained spectra for membranes soaked in the Wheel solution were similar to those obtained for the Insect solutions (Figure 13). However, significant differences in the morphology of the membrane surfaces were revealed using microscopic examination.

**Figure 14:**
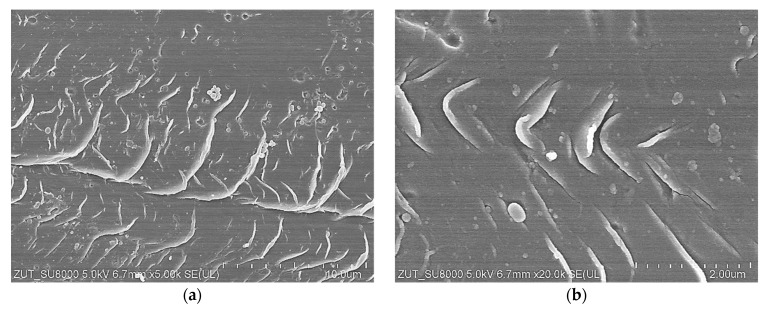
SEM images of UE50 membrane surface after 18 months of soaking time w a 0.5% Insect solution: (**a**) 5k magnification and (**b**) 20k magnification.

SEM examinations showed that the surface images of UE10 membranes did not change significantly after 18 months and were similar to those presented after 5 months of soaking (Figure 10b). The local linear distortions shown in Figure 10d were not observed on the tested samples. However, the results of the observations of UE50 membranes were different. In this case, the surface was covered with numerous structures resembling “fish scales” (Figure 14). The amount of spherical PVP agglomerates (Figure 10e) decreased significantly, and there was not as much surface damage, as shown in Figure 10g.

**Figure 15:**
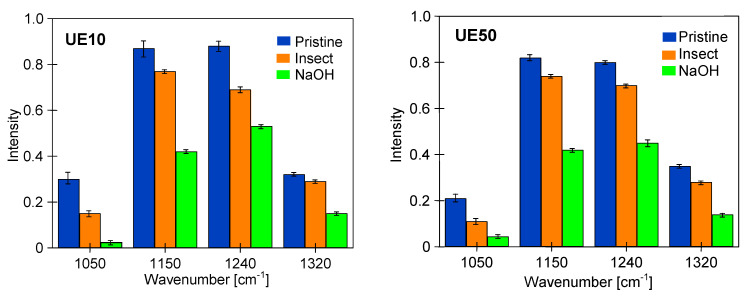
Intensity changes of peaks from FTIR spectra obtained for pristine commercial membranes and these membranes soaked for 18 months in a 0.5% Insect solution and for 7 days in 1 M NaOH solution. FTIR measurements were performed for five membrane samples.

The analysis of the FTIR test results indicates that the changes in the membrane surface observed in SEM images resulted from the cracking of polymer chains, which is caused by the impact of NaOH [1,14,24,27]. The degradation effect visible on the FTIR-ATR spectrum is the gradual disappearance of the peak characteristics for the PES skeleton, such as C-O-C stretching vibrations (1050 cm^−1^), Ar–SO_2_–Ar symmetric stretching vibrations (1150 cm^−1^), Ar–O –Ar stretching vibrations (1240 cm^−1^) and Ar–SO_2_–Ar asymmetric stretching vibrations (1320 cm^−1^) [6,14,20]. Changes in the intensity of these peaks are shown in Figure 15. The results obtained indicate that soaking the membrane for 7 days in a 1 M NaOH solution (pH = 14) caused greater degradation than during 18 months of contact with the Insect solution (pH = 11.5). This result confirms the manufacturers’ indications that PES membranes can separate solutions with a pH below 12 [6,14,19]. 

**Figure 16:**
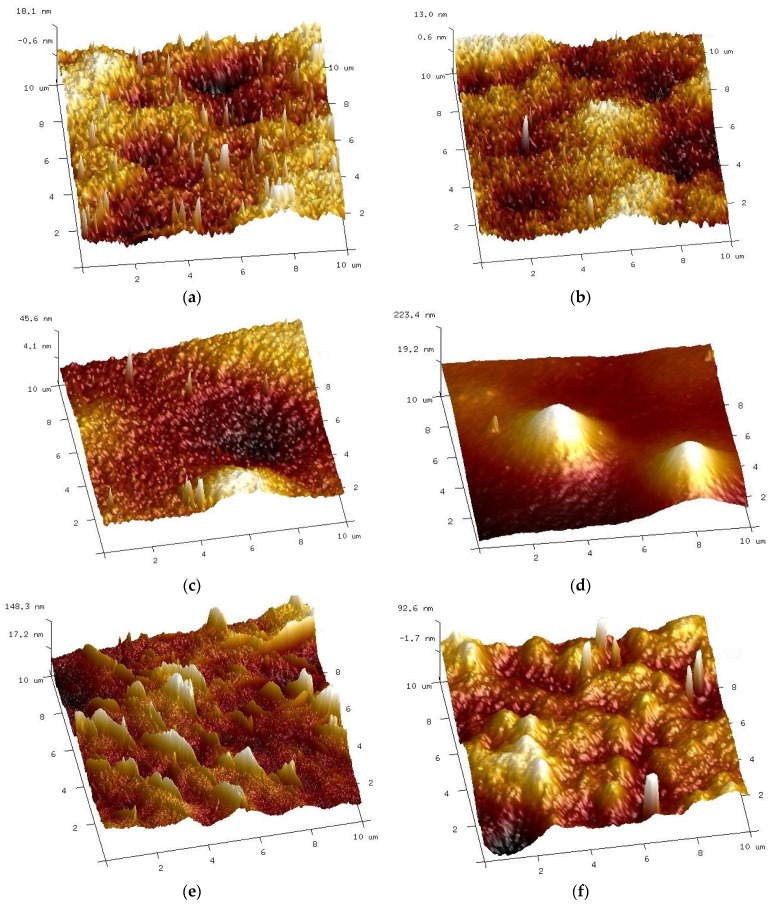
AFM of surface PES membranes after 18 months of soaking in a 0.5% Insect solution ((**a**)—self-made PES, (**c**)—UE10 and (**e**)—UE50) and in a 0.5% Wheel solution: (**b**) self-made PES, (**d**) UE10 and (**f**) UE50.

AFM studies showed that changes in the surface structure depended on both the type of membranes and the alkaline agent used. Compared with the pristine membranes (Figure 8a), the smallest changes were found for self-made PES membranes (Figure 16a,b). In this case, similar surface images were obtained for both samples soaked in Insect and Wheel solutions. The action of these solutions resulted in a more than four-fold reduction in roughness; for example, parameter Rq decreased from 23.5 to 5.4–6.1 nm (Table 4). 

During the experimental investigation, it was observed that the surface of commercial membranes was changed to a greater extent by the Wheel solution. Insect treatment caused the smooth surface of the UE10 membrane (Figure 11a) to acquire a coarse-grained structure (Figure 16c). The presence of conical bulges of considerable height was found on samples taken from the Wheel solution (Figure 16d). As a result, the roughness of the UE10 membranes increased, e.g., Rq from 15.2 to 27.6 nm (Insect) and 47.9 nm (Wheel). In the case of the UE50 membrane, the changes in roughness were smaller, and larger changes were caused by the Insect solution; for example, the Rq parameter increased from 21.3 to 28.8 nm. There were numerous thin protruding structures on the surface, resembling “fish scales”, which were also observed during SEM examinations (Figure 14). It must be recognized that the surface parameters for the UE50 membrane soaked in Wheel liquid were similar to those obtained for pristine membranes (Table 4). This was probably the reason for the improved degree of dextran retention (Figure 12, UE50).

### 3.4. Membrane Performance after 18 Months of Soaking Time

**Figure 17:**
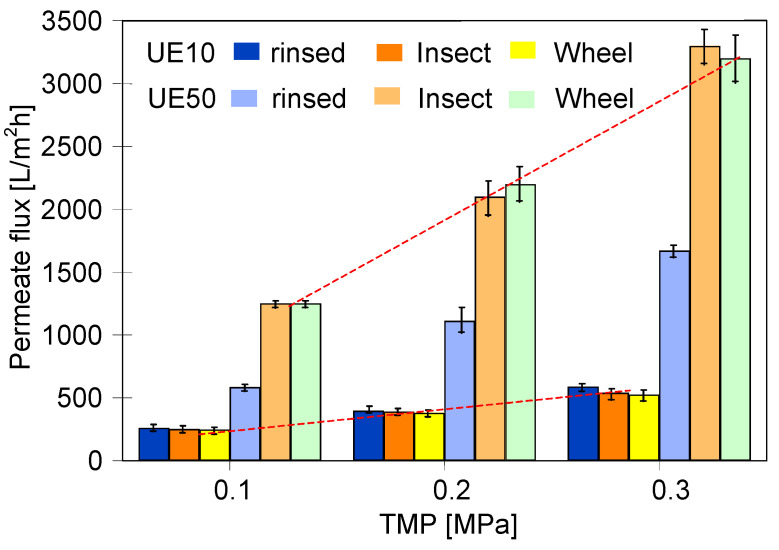
The influence membranes soaking (18 months) in the 0.5% alkaline solutions (Insect and Wheel) on the maximum permeate flux. Feed—distilled water.

The result of soaking in alkaline solutions was the enlargement of the pores in PES membranes, and as a result, a significant increase in the permeate flux was observed [19]. An increase in permeate flux as a result of membranes soaking in alkaline solutions was also found in the conducted research, especially for the UE50 membrane. Commercial membranes contain preservatives that block pores. Therefore, the initial maximal permeate flux was determined after 2 h of rinsing the pristine membranes in the 0.5% Insect solution (Figure 17, rinsed). As shown by the FTIR tests with NaOH solution (Figure 4), such a short period did not cause any changes in the composition of the membrane matrix. After this operation, the efficiency of UE10 was 394 L/m^2^h (TMP = 0.2 MPa), and after 18 months of soaking, it slightly decreased to 390 (Insect) and 380 L/m^2^h (Wheel). This was most likely due to changes in the density of the structure, which resulted in a smoother surface (Figure 16, UE10).

It is essential to mention that a different result was observed for the UE50 membrane. In this case, the initial performance of 1100 L/m^2^h (TMP = 0.2 MPa) after 18 months of soaking increased to 2100 L/m^2^h (Insect) and 2200 L/m^2^h (Wheel). This result was confirmed by microscopic images showing numerous large changes in the surface structure (Figure 16e,f). Moreover, the UE50 membranes contained more PVP in their structure (Figure 10); hence, their leaching by alkaline solutions simultaneously increased the membrane permeability. The permeate flux increases due to the degradation of PES membranes by NaOH solutions, which enlarges the pore size [19]. This was attributed to the pore size increase at higher pH values [9,19]. Similar effects were found for the polyamide selective layer, which was attributed to the NaOH-catalyzed hydrolysis, leading to the breakage and removal of some polyamide chains [28].

An important point that should be noted is that porous structures can compress under the influence of increasing pressure, causing non-linear changes in the liquid flow. However, in the examined case, an increase in the TMP value resulted in a linear increase in the permeate flux (Figure 17, broken lines). This noteworthy result indicates that changes in the structure of the surface layer did not reduce its stability.

**Figure 18:**
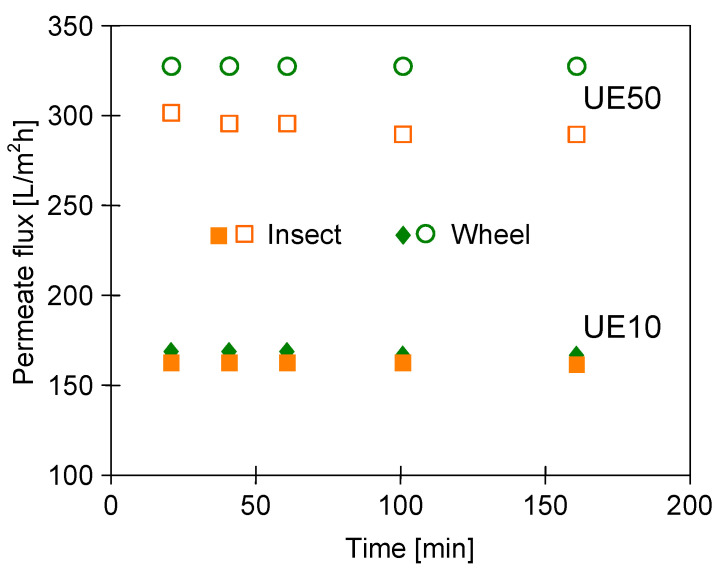
The influence membranes soaking (18 months) in the 0.5% alkaline solutions (Insect and Wheel) on the permeate flux. Feed—synthetic wastewater.

The decrease in the dextran rejection value (Figure 12) and the increase in permeate flux (Figure 17) indicate that the separation properties of the membranes deteriorated after 18 months of soaking. However, in addition to the membrane performance, the separation efficiency in the UF process was also influenced by the polarization layer created by the feed components. For this reason, in the last stage of the research, it was checked how membranes soaked for 18 months in alkaline solutions separate the wastewater generated during car washing. Synthetic wastewater, which is a mixture of surfactants (Turbo Foam) and hydrowaxe, was used for the tests. The obtained permeate flux was much smaller than the values obtained for distilled water as a feed (Figure 18). For the UE10 membranes, the flux decreased from 245 to 161 L/m^2^h (Insect) and to 165 L/m^2^h (Wheel). A much greater influence of the alkaline agent type was found for the UE50 membranes. Indeed, in this case, the flow decreased from 1250 to 300 L/m^2^h (Insect) and to 326 L/m^2^h (Wheel). During 160 min of the UF process run, the obtained process performance was stable; only in the case of UE50 (Insect), the permeate flux decreased from 300 to 288 L/m^2^h. The reduction in the initial permeate flux indicates that during the first minutes of the UF process, the feed components, mainly fine suspension (NTU 4.8), filled the larger pores, which reduced the permeate flow. The observed subsequent stabilization of the flux values indicates that membrane fouling did not increase in the following minutes.

**Figure 19:**
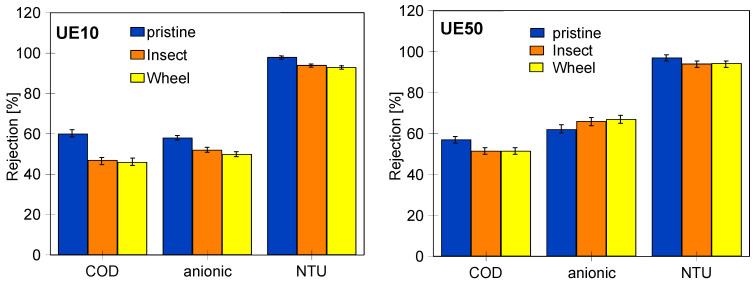
Changes in the COD, anionic surfactant and NTU rejection during UF process of synthetic wastewater.

Despite the observed significant changes in the structure of the outer layer after 18 months of soaking the membranes, the obtained rejection rate of the components of the tested mixture was surprisingly high and similar to the values obtained for pristine membranes (Figure 19). For UE10 membranes, the COD rejection value decreased from 60% to 47 (Insect) and 46% (Wheel). The membranes still separated suspended solids well, and the NTU rejection decreased from 98% to only 94% (Insect) and 93% (Wheel). The UE50 membranes showed equally good separation. For the COD parameter, rejection decreased from 57 to 51.5%, and for NTU, it decreased from 97 to 94%. In both cases, high-quality permeate with a turbidity of 0.18–0.25 NTU was obtained.

The results obtained for the separation of the components of the cleaning agent mixture are much better than the results for the dextran separation. These differences are ascribed to the formation of a stagnant, highly concentrated layer near the membrane surface due to consolidation and aggregation of solute [31]. This outcome may indicate that fouling and aggregation effects outweigh the physical and chemical changes that take place in the membranes during chemical treatments, which was also observed in work [24]. Moreover, the presented results confirmed that the PVP leakage from the membrane matrix seems to have no impact on membrane retention if the fouling layer occurs [1].

To be complete, it should be noted that the influence of the solutes and polarization layer properties on the rejection degree was also visible for pristine membranes. The UE10 (10 kDa) membranes, having much smaller pores than the UE50 (100 kDa) membranes, retained dextran to a greater extent (Figure 12). Meanwhile, similarly high rejection values were obtained during the synthetic wastewater separation (Figure 18). Studies focused on the separation of similar wastewater types showed that, in this case, the formation of the cake layer on the membrane surface was the dominant fouling mechanism [11].

## 4. Conclusions

This research showed that alkaline cleaning solutions can cause significant changes in the structure of ultrafiltration PES membranes, especially if they contain PVP additives. It was confirmed that long-term contact of membranes with NaOH solutions causes PVP to be washed out from the membrane matrix structure. As a result, the pores were enlarged, resulting in a significant increase in maximal permeate flux and a 30–60% reduction in dextran rejection. Greater resistance to NaOH and, hence, better separation was achieved for membranes made of pure PES.

The evidence from this study demonstrates that PVP added to the membrane does not mix well with PES and may form agglomerates inside the separation layer. Such places in the membrane are weakened, facilitating their degradation under the influence of alkaline solutions. The size of the damage occurring on the membrane surface may exceed several micrometers.

The results obtained indicate that PES membranes with the addition of PVA are not recommended for continuous separation of solutions containing NaOH with a pH value over 11. However, the high resistance of the tested membranes to shorter exposure times of NaOH solutions indicates that alkaline solutions can be used for periodic cleaning of fouled PES membranes.

Finally, despite significant changes in structure, the tested PES membranes, after 18 months of soaking in alkaline solutions during wastewater separation, showed rejection solutes similar to pristine membranes. The main conclusion is that PES membranes can be used to treat car wash wastewater, even if their alkaline components cause minor damage to the separation layer.

## Figures and Tables

**Table 1:** The composition of the cleaning agents concentrates.

Name	Component	Concentration [%]
Wheel	sodium hydroxide	3–5
	ethylenediaminetetraacetic Acid Tetrasodium Salt	3–5
	Sulfonic Acids, C14-16-Alkane Hydroxy and C14-16-Alkene, Sodium Salts	5–7
	Diethylene glycol butyl ether	3–5
Insect	1-Propanaminium, 3-amino-N-(carboxymethyl)-N,N-dimethyl-, N-(C12-18(even numbered) acyl) derivs	0.5–1.5
	sodium hydroxide	3–5
	Sulfonic Acids, C14-16-Alkane Hydroxy and C14-16-Alkene, Sodium Salts	5–7
	Diethylene glycol butyl ether	3–5
	ethylenediaminetetraacetic Acid Tetrasodium Salt	3–5

**Table 2:** Characteristic peaks/bands observed during FTIR examination of PES membranes and PVP.

Peak No.	Frequency [cm^−1^]	Functional Groups	References
1	717	C-S	[10]
2	700–831	Aromatic sulfone	[14]
3	1045–1055	C-O-C stretching vibrations	[14,21]
4	1103	S=O	[14]
5	1149	C-O stretching vibration	[6]
6	1239–1240	C-O-C stretching vibrations	[6,14]
7	1298	C-O-C stretching vibrations	[6]
8	1320–1322	–SO^2−^ stretching vibration	[6,14]
9	1386	C-N (PVP)	[20]
10	1484	C-S	[6]
11	1576	aromatic systems	[6]
12	1560–1597	C=C ring vibration	[1,14]
13	1652	C=O	[20]
14	1660	C=C stretching vibration	[6]
15	1635–1695	C=O amide bound (PVP)	[1,14,19]
16	1710	C=O stretching vibration	[27]
17	2800–2900	vibrations –CH_2_	[10]

**Table 3:** The results of SEM-EDS analysis.

Element	C	O	S	Na
UE10	54.39	36.81	8.72	0.08
UE10 rinsed	61.02	31.77	7.06	0.15
UE50	50.35	43.72	5.57	0.36
UE50 rinsed	49.87	42.34	6.86	0.93

**Table 4:** AFM studies—changes the roughness parameters (Rq and Ra).

Membrane	Rq [nm]	Ra [nm]
**PES**		
pristine	23.5 ± 3.3	19.1 ± 3.2
Insect	6.1 ± 0.6	4.7 ± 0.5
Wheel	5.4 ± 0.6	4.1 ± 0.7
**UE10**		
pristine	15.2 ± 2.7	12.5 ± 2.1
Insect	27.6 ± 9.7	19.7 ± 6.4
Wheel	47.9 ± 8.6	30.3 ± 6.2
**UE50**		
pristine	21.3 ± 1.6	16.9 ± 1.1
Insect	28.8 ± 6.2	22.6 ± 4.7
Wheel	20.4 ± 3.9	16.3 ± 2.9

## Data Availability

Data are contained within the article.
